# Necrosensor: a genetically encoded fluorescent sensor for visualizing necrosis in *Drosophila*

**DOI:** 10.1242/bio.060104

**Published:** 2024-01-22

**Authors:** Hiroshi Nishida, Antonio Bolea Albero, Kenta Onoue, Yuko Ikegawa, Shivakshi Sulekh, Ugurcan Sakizli, Yasuhiro Minami, Shigenobu Yonemura, Yu-Chiun Wang, Sa Kan Yoo

**Affiliations:** ^1^ Division of Cell Physiology, Graduate School of Medicine, Kobe University, Kobe, 650-0017, Japan; ^2^ Physiological Genetics Laboratory, RIKEN CPR, Kobe, 650-0047, Japan; ^3^ Laboratory for Epithelial Morphogenesis, RIKEN BDR, Kobe, 650-0047, Japan; ^4^ Laboratory for Ultrastructural Research, RIKEN BDR, Kobe, 650-0047, Japan; ^5^ Laboratory of Molecular Cell Biology and Development, Kyoto University, Kobe, 650-0047, Japan; ^6^ Laboratory for Homeodynamics, RIKEN BDR, Kobe, 650-0047, Japan; ^7^ Division of Developmental Biology and Regenerative Medicine, Graduate School of Medicine, Kobe University, Kobe, 650-0047, Japan; ^8^ Department of Cell Biology, Tokushima University Graduate School of Medicine, Tokushima, 770-8503, Japan

**Keywords:** *Drosophila*, Necrosis, Necrosis sensor, Cell death, *In vivo*

## Abstract

Historically, necrosis has been considered a passive process, which is induced by extreme stress or damage. However, recent findings of necroptosis, a programmed form of necrosis, shed a new light on necrosis. It has been challenging to detect necrosis reliably *in vivo*, partly due to the lack of genetically encoded sensors to detect necrosis. This is in stark contrast with the availability of many genetically encoded biosensors for apoptosis. Here we developed Necrosensor, a genetically encoded fluorescent sensor that detects necrosis in *Drosophila*, by utilizing HMGB1, which is released from the nucleus as a damage-associated molecular pattern (DAMP). We demonstrate that Necrosensor is able to detect necrosis induced by various stresses in multiple tissues in both live and fixed conditions. Necrosensor also detects physiological necrosis that occurs during spermatogenesis in the testis. Using Necrosensor, we discovered previously unidentified, physiological necrosis of hemocyte progenitors in the hematopoietic lymph gland of developing larvae. This work provides a new transgenic system that enables *in vivo* detection of necrosis in real time without any intervention.

## Teaser

**Summary:** This paper describes the development of a new genetically encoded necrosis sensor in *Drosophila*.

## INTRODUCTION

Cell death is an indispensable process for the lifecycle of organisms. There are three major types of cell death based on pathological features: apoptosis, autophagic cell death and necrosis. For a long time, necrosis has been categorized as accidental cell death, defined by the absence of the features of apoptosis and autophagy (Doherty and Baehrecke, 2018; Edinger and Thompson, 2004; Galluzzi et al., 2018). Necrosis occurs when the plasma membrane is breached or when cellular energy levels become too low to maintain life. During necrosis, an influx of water causes organelles to enlarge, leading to cell swelling and lysis. Recent findings of necroptosis, necrosis that is molecularly regulated by TNF-mediated activation of RIPK-MLKL signaling, sparked interest in this field (Galluzzi et al., 2018; Khan et al., 2014; Pasparakis and Vandenabeele, 2015).

Detecting cell death is crucial for understanding its molecular mechanisms. The development of various tools to detect apoptosis has paved the way for significant advances in our understanding of the process. These tools include non-genetic methods such as detection of cleaved caspase and TUNEL staining (Arama and Steller, 2006; Denton and Kumar, 2015; Sarkissian et al., 2014), although TUNEL is not strictly specific to apoptosis (Ciesielski et al., 2022; de Torres et al., 1997; Grasl-Kraupp et al., 1995; Yang et al., 2014). A range of genetically encoded biosensors that detect apoptosis in organisms *in vivo* have also been generated: SCAT (Takemoto et al., 2003), CIETDY reporter (Mazzalupo and Cooley, 2006), CD8-PARP-Venus (Florentin and Arama, 2012; Williams et al., 2006), Apoliner (Bardet et al., 2008), CaspaseTracker (Tang et al., 2015), iCasper (To et al., 2015), CasExpress (Ding et al., 2016), GC3Ai (Schott et al., 2017) and DBS (Baena-Lopez et al., 2018). These genetically encoded biosensors have contributed tremendously to *in vivo* apoptosis research.

In contrast, no genetic biosensor has been available to detect necrosis *in vivo* in any organism. To detect necrosis in tissue culture *in vitro*, ELISA-based quantification of secreted cytoplasmic or nuclear components such as lactate dehydrogenase or HMGB1 is often used (Kepp et al., 2011; Martins et al., 2013). However, this is not compatible with *in vivo* situations. The only method to detect necrosis *in vivo* to date is utilization of vital dyes, such as Propidium Iodide (PI) (Li et al., 2019; Obata et al., 2015; Yang et al., 2013) and sytox (Kolahgar et al., 2015; Liang et al., 2017). These vital dyes enter necrotic cells upon disruption of the plasma membrane and bind to nucleic acids. However, the use of these vital dyes presents several problems. They frequently have high background signals in the cytoplasm due to RNA, which is independent of necrosis. Vital dyes cannot enter nuclei of some type of cells. This method also requires sacrificing animals and careful live staining. It is important to note that tissues can start to undergo necrosis during dissection and live staining, confounding interpretation of these vital dye-based observations. Moreover, some vital dyes such as sytox are not compatible with fixation either (Kolahgar et al., 2015; Liang et al., 2017), necessitating a speedy procedure of live imaging immediately after live staining. PI could also interfere with the fluorescence from other fluorophores (Stokke et al., 1998). The complications of the current necrosis detection methods could hinder the progression of necrosis research. To overcome these technical issues, we have created the first transgenic system with a genetically encoded necrosis sensor in *Drosophila*, which can detect necrosis *in vivo* without the necessity of live staining or, potentially, sacrifice of animals.

## RESULTS

**Fig. 1.**
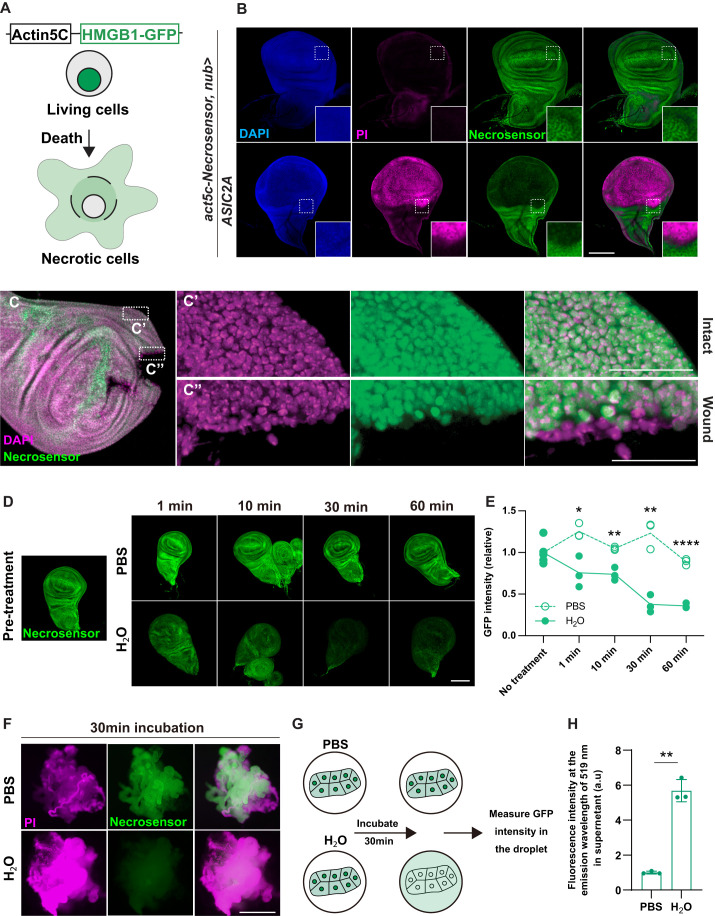
**Necrosensor detects necrosis induced by various stresses.** (A) Design of Necrosensor. Necrosensor is localized in the nucleus of healthy cells. Upon necrosis, it is secreted to the extracellular space. (B) Necrosensor is excluded from necrotic cells when necrosis is induced by ASIC2A CA-mediated sodium influx in the wing pouch. Note an inverse pattern of Necrosensor and PI. nub-Gal4 is a wing pouch driver. The dashed rectangles are magnified at the bottom right of each image. (C) A wing disc was injured *ex vivo* with tweezers. Necrosensor detects wounding-induced necrosis. The areas in the dashed rectangles are magnified in the images on the right. (D,E) Wing discs were dissected out and cultured in either PBS or H_2_O for 1, 10, 30 or 60 min. The GFP intensity within whole wing disc at each time point was measured. Two-tailed unpaired *t*-test. (F) Larvae expressing Necrosensor were dissected in PBS, followed by being cultured either with PBS or H_2_O for 30 min. (G) A schematic of the experiment conducted in Fig. 1H. (H) GFP signals of Necrosensor released from the fat body cells into the culture supernatant was detected by nanodrop. Two-tailed unpaired *t*-test. Scale bars: 100 μm (B,D); 25 μm (C); 500 μm (F).

We decided to use the nuclear protein HMGB1 (high-mobility group box 1) as the basis of our necrosis sensor, because HMGB1 is passively released during necrosis in tissue culture (Scaffidi et al., 2002). This secreted HMGB1 can function as a DAMP to induce immune responses (Klune et al., 2008; Raucci et al., 2007; Yanai et al., 2012). Detection of released HMGB1 in the extracellular space is commonly used as a marker for necrosis in tissue culture *in vitro* (Kepp et al., 2011; Martins et al., 2013). We generated a stable transgenic line that expresses HMGB1-GFP under an actin5c enhancer (Fig. 1A). Using this ubiquitous expression enhancer enables us to combine the sensor with the GAL4-UAS system. The transgenic flies with a copy of actin5c-HMGB1-GFP developed normally, were fertile, and were physically indistinguishable from their wild-type siblings. We named this genetic biosensor Necrosensor. We examined whether Necrosensor can accurately detect necrosis in a variety of circumstances. If Necrosensor detects necrosis authentically, it should lose the nuclear localization in necrotic cells, displaying an inverse pattern with PI staining.

We first induced necrosis by ectopically expressing constitutively active sodium channel ASIC2A (ASIC2 CA) in the wing imaginal disc (Fig. 1B), since ASIC2A induces necrosis through a constitutive influx of sodium in the fly brain (Liu et al., 2013). We verified, by electron microscopy (EM), that expression of *ASIC2A CA* in the wing pouch induces typical morphological features of necrosis, such as severely disrupted nuclear and cellular membranes and decreased electron staining, whereas acute expression of the potent apoptosis inducer *reaper* (*rpr*), which suppresses caspase inhibitor DIAP1 (Goyal et al., 2000) showed neither of them (Fig. S1A). miRNA for *rpr*, *hid* and *grim* (Siegrist et al., 2010) did not suppress ASIC2A-mediated wing destruction, which is in a contrast with its suppression of rpr-induced lethality (Fig. S1B). Consistent with EM images, PI entered the cytoplasm of ASIC2A-induced necrotic cells. Inversely, Necrosensor signals were lost in the area that expresses *ASIC2A CA* (Fig. 1B). These data demonstrate that Necrosensor detects necrosis that is induced by ASIC2A-mediated sodium influx.

Next, we examined whether Necrosensor can detect necrosis induced by injury. Severe damage, such as physical wounding, induces cell death in tissues (Edinger and Thompson, 2004; Galluzzi et al., 2018; Yoo et al., 2016). When we physically wounded the wing imaginal disc, Necrosensor detected necrotic cells adjacent to the wound, but not elsewhere (Fig. 1C). Importantly, the cells that lost Necrosensor at the wound edge did not demonstrate cDCP1 staining (Fig. S1C).

In addition to wounding by physical injury, we also employed X-ray irradiation to induce DNA double-strand breaks and oxidative stress, which results in apoptosis and necrosis (Nakano and Shinohara, 1994; Zhao et al., 2019). We irradiated L3 larvae to induce cell death in the wing imaginal discs. Necrosensor detected X ray-induced necrosis and exhibited an inverse pattern with PI staining (Fig. S1D).

We also tested Necrosensor's ability to detect oncogene-induced necrosis. Oncogenic stress, which is induced by oncogene expression, causes apoptosis and necrosis in addition to cell proliferation (Hanahan and Weinberg, 2011; Nishida et al., 2021). The extent of tumor necrosis is correlated with aggressive symptoms and chemotherapy responsiveness (Bredholt et al., 2015; Hanahan and Weinberg, 2011; Nishida et al., 2021). In *Drosophila*, oncogenes such as *src* and *ras* induce cell death in addition to cell proliferation (Karim and Rubin, 1998; Nishida et al., 2021; Pedraza et al., 2004). We expressed constitutively active *Src42A*, a Src family kinase, in a localized region of the wing imaginal disc. *Src42A CA* expression disrupted the wing imaginal tissue organization. In the disorganized area, necrosis was detected by Necrosensor (Fig. S1E).

We further tested whether Necrosensor recapitulates the known behavior of HMGB1 during necrosis. Extracellularly released HMGB1 from necrotic cells is used as a marker of necrosis in mammals. (Galluzzi et al., 2018; Kepp et al., 2011). Thus, we examined whether Necrosensor can also be detected in the extracellular space after necrotic stimuli. We decided to induce necrosis acutely through osmotic stress by culturing the larval tissues *ex vivo* with water and to measure secreted Necrosensror in water. Upon necrosis induction with H_2_O, Necrosensor was lost from the wing disc (Fig. 1D,E) or the larval carcasses which included the gut, salivary gland and fat body (Fig. 1F). To examine whether Necrosensor is secreted to the extracellular space, we used the fat body, which comprises one of the largest cells in the larva and contains a large amount of Necrosensor. This feature of the fat body would make detection of secreted Necrosensor more sensitive. We found that culturing the fat body with water but not with PBS induces secretion of Necrosensor to the culture water (Fig. 1G-H). This indicates that Necrosensor lost from the cell nuclei is released into the extracellular space, consistent with the previously reported behavior of HMGB1 (Galluzzi et al., 2018; Kepp et al., 2011).

**Fig. 2.**
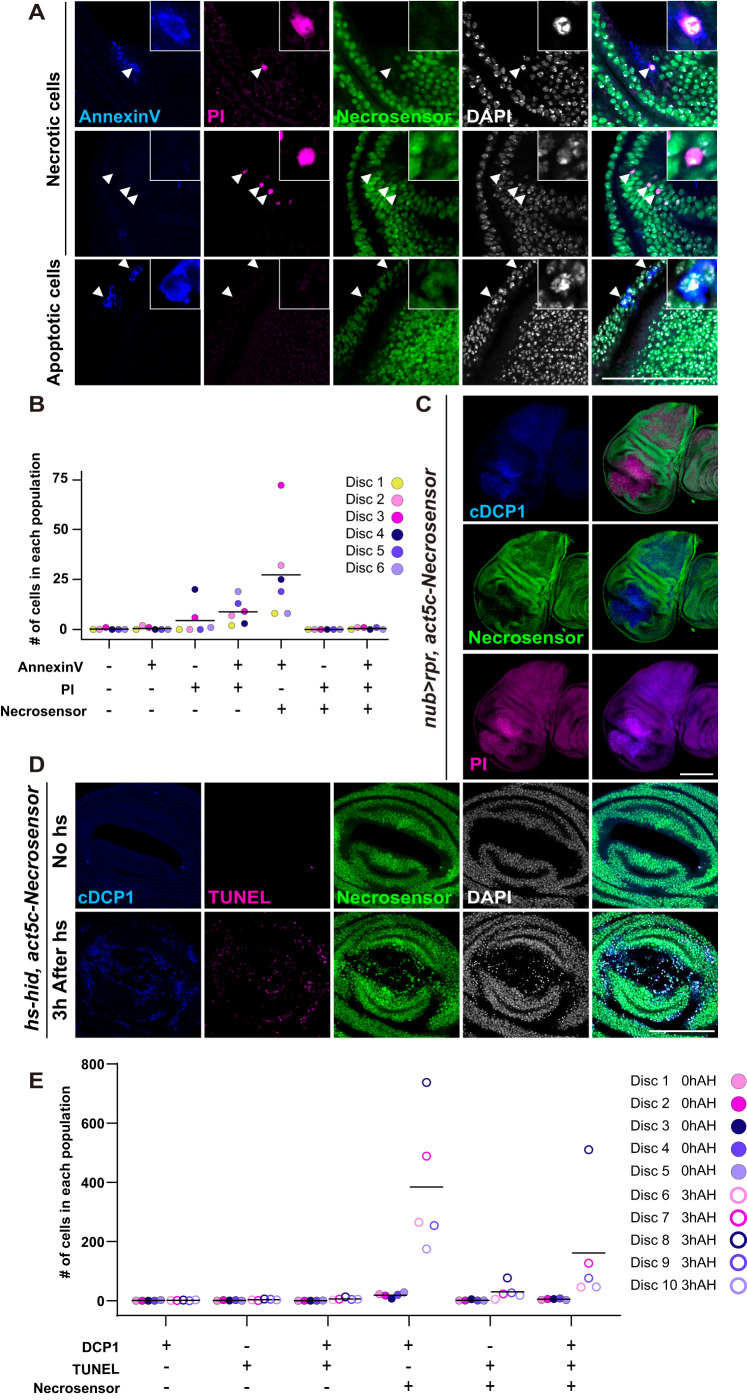
**Characterization of Necrosensor.** (A) Wing discs were dissected out followed by staining with Annexin V and PI. Arrowheads indicate cells undergoing multiple types of cell death. (B) Physiologically dying cells in the wing disc were categorized into seven groups. Six independent wing discs were used for the analysis. Annexin V+, PI+ and Necrosensor- indicate that cells are stained by Annexin V, are labeled by PI and have lost Necrosensor, respectively. (C) Apoptosis induction by rpr leads to disappearance of Necrosensor, likely due to secondary necrosis. (D,E) Apoptosis was induced via a short pulse expression of *hid* to observe Necrosensor localization in early apoptotic cells. At 3 h after hid induction, apoptotic cells marked by cDCP1 and TUNEL appear. These cells still have Necrosensor in the nucleus. DCP1+, TUNEL+ and Necrosensor- indicate that cells have cDCP1, are labeled by TUNEL staining and have lost Necrosensor, respectively. Scale bars: 100 μm (A,C,D).

HMGB1's behavior has been extensively characterized in mammalian systems, and our data above support that Necrosensor behaves in a similar manner. To further validate whether Necrosensor detects necrosis faithfully, we compared Necrosensor and known cell death markers. We took advantage of physiological cell death that spontaneously occurs in the wing disc (Shinoda et al., 2019; Zhang et al., 2015). This setting enables simultaneous detection of various types of cell death. Dissected wing imaginal discs were stained with Annexin V and PI simultaneously, which is a conventional way to categorize the state of dying cells (Vitak et al., 2015). Annexin V is a protein that binds to phosphatidylserine, an early apoptosis marker. Exposure of phosphatidylserine on the cell surface is an ‘eat me’ signal, which recruits macrophages (Segawa and Nagata, 2015). However, when apoptotic cells are not completely cleared, these cells lose the integrity of the plasma membrane, leading to secondary necrosis (Galluzzi et al., 2018). Cells that undergo secondary necrosis have features of both apoptosis (condensed chromatin) and necrosis (a ruptured plasma membrane). It was originally thought that HMGB1 was not released during secondary necrosis (Scaffidi et al., 2002), but subsequent studies have demonstrated that HMGB1 is indeed secreted from cells undergoing secondary necrosis (Bell et al., 2006; Choi et al., 2004; Martins et al., 2013; Obeid et al., 2007; Tian et al., 2007). In addition to apoptotic cells, Annexin V also labels necrotic cells because Annexin V can bind to the phosphatidylserine in the inner side of necrotic cells. Thus, it is well accepted that Annexin V (A) +, PI- indicates apoptosis; A+, PI+, necrosis (either primary or secondary necrosis); A-, PI+, early necrosis, since PI is smaller than the Annexin V protein. If Necrosensor detects necrosis faithfully, it should label A+, PI+ cells and A-, PI+ cells, but not A+, PI- cells. We found that necrotic cells (A+, PI+ or A-, PI+) lose Necrosensor while apoptotic cells (A+, PI-) do not lose Necrosensor (Fig. 2A,B). This indicates that Necrosensor faithfully detects necrosis.

Furthermore, we compared Necrosensor to DCP1 activation and TUNEL staining. Dcp-1 is an executioner caspase (Song et al., 1997). TUNEL detects DNA fragmentation, which is expected to occur either after caspase activation during apoptosis, or during other cell death such as necrosis (Ciesielski et al., 2022; de Torres et al., 1997; Grasl-Kraupp et al., 1995; Yang et al., 2014). Thus, cDCP1+, TUNEL- is early apoptosis, cDCP1+, TUNEL+ is either late apoptosis or secondary necrosis, and cDCP1-, TUNEL+ is primary necrosis. We found that Necrosensor is retained in early apoptotic cells (cDCP1+, TUNEL-) (Fig. S2A-B). Among cDCP1+, TUNEL+ (either late apoptosis or secondary necrosis), we found that more cells retain Necrosensor, which are likely late apoptotic cells, not yet undergoing secondary necrosis. Regarding DCP1-, TUNEL+ necrotic cells, more cells retained Necrosensor, suggesting that DNA fragmentation occurs earlier than the membrane breach detected by Necrosensor (Fig. S2A,B).

We also applied triple labeling by AnnexinV (A), PI and Necrosensor to irradiated wing discs. Consistent with the physiological cell death context, with irradiation, which induces necrosis more actively, we found that apoptotic cells (A+, PI-) do not lose Necrosensor (Fig. S3A,B). Among necrotic cells (A+, PI+), we found that a majority of cells also lose Nercosensor but some retain it (Fig. S3A,B). Although PI and Necrosensor are expected to behave in a similar manner, PI enters cells slightly earlier than loss of HMGB1, likely due to the size difference of the small molecule PI and the protein HMGB1.

In addition to physiological cell death and radiation, we extended our detailed analyses to apoptosis-induced secondary necrosis. We speculated that generating a large quantity of apoptotic cells, which exceeds the phagocytosing ability of hemocytes’, should give rise to secondary necrotic cells. We induced massive apoptosis in a large area of the wing disc by expressing *rpr*. As we predicted, Necrosensor detected secondary necrosis that ensued apoptosis (Fig. 2C), which is consistent with previous studies *in vitro* (Bell et al., 2006; Choi et al., 2004; Martins et al., 2013; Obeid et al., 2007; Tian et al., 2007). To prove that Necrosensor is detecting secondary necrosis following rpr-induced apoptosis, not apoptosis itself, we induced apoptosis by a short pulse expression of *hid,* which acts similarly to *reaper* to potently inhibit DIAP1 (Goyal et al., 2000) and performed triple labeling of cDCP1/TUNEL/Necrosensor 3 h after *hid* induction. In this case, early apoptotic cells (DCP1+, TUNEL-) cells did not lose Necrosensor (Fig. 2D,E). These observations support the idea that Necrosensor can distinguish apoptosis, and secondary necrosis following apoptosis.

**Fig. 3.**
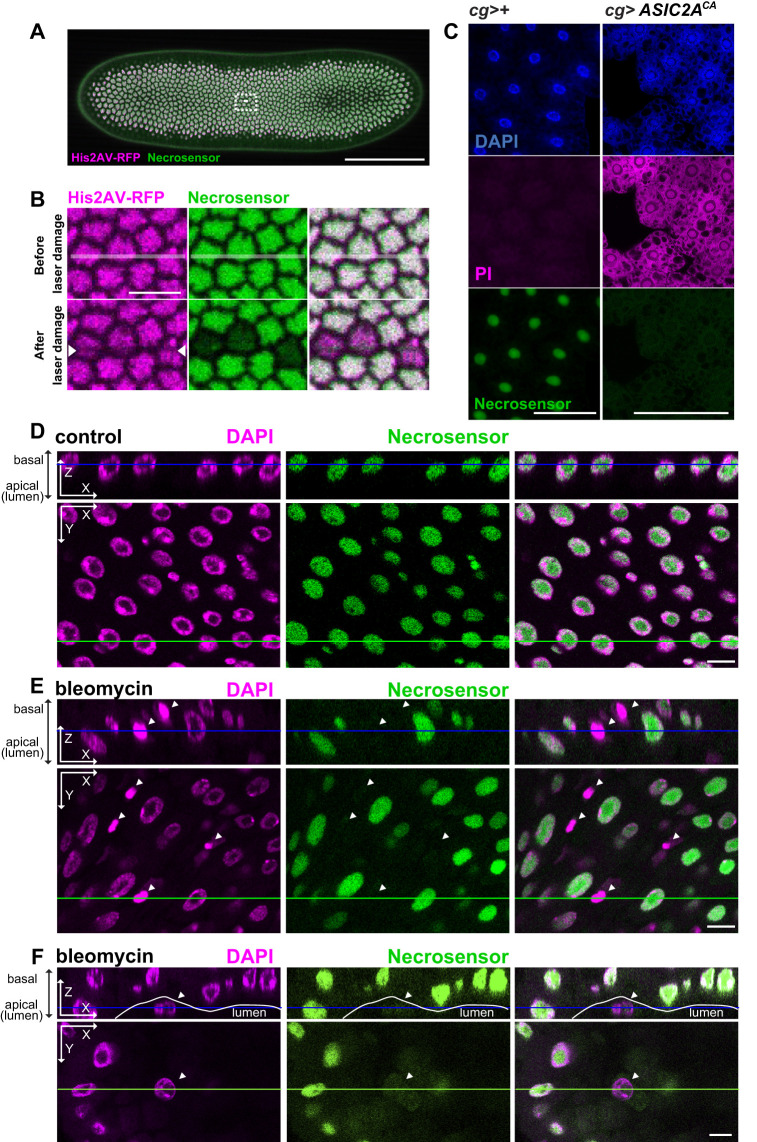
**Necrosensor detects necrosis in diverse tissues. (**A) Single Z slice image of a representative Necrosensor (green)/His2Av-RFP (magenta) embryo imaged on the dorsal side during cellularization prior to laser damage. The rectangle with white dashed lines indicates the region shown in B. (B) Necrosensor detects laser-inflicted damage of embryonic cells within 1 s after the damage. The laser line scan was performed along the lines overlaid over the ‘before laser damage’ images. Note that His2AV-RFP signals were lost sharply only in the ablated area due to photobleaching (arrowheads) but that Necrosensor signals disappeared from the whole nuclei. (C) Necrosensor detects sodium influx-mediated necrosis in the fat body. (D–F) Necrosensor detects necrosis of the gut cells induced by bleomycin treatment. Necrotic cells are observed in both the tissue layer (E) and the gut lumen (F). Arrowheads indicate necrotic cells. Scale bars: 100 µm (A); 10 µm (B); 50 µm (C); 10 µm (D–F).

To further assess the versatility of Necrosensor, we examined its ability to detect necrosis in various tissues. First, we inflicted damages on early embryonic cells using a pulsed infrared (IR) laser, followed by live imaging analysis. We reasoned that a laser-inflicted cellular damage would induce necrosis. As expected, immediately following (<1 s) the single line scan of the IR laser, Necrosensor was excluded from the nucleus (Fig. 3A,B). Importantly, the RFP-tagged Histone H2A variant (His2Av-RFP) was retained in the cells after the damage, suggesting that not all nuclear proteins are excluded from the nucleus during necrosis. We confirmed that among 153 damaged cells in three embryos, only one cell divided at a later time point, suggesting that the cells damaged by IR laser were dead, or at least not metabolically active. These data demonstrate that Necrosensor can be used in live embryos.

Necrosis of adipose tissues is a clinically important hallmark of obesity (Cinti et al., 2005). Thus, in addition to the H_2_O-induced necrosis above (Fig. 1F–H), we induced necrosis by ASIC2A CA-mediated sodium influx in the fat body. The nuclear localization of Necrosensor was lost upon expression of ASIC2A CA, demonstrating the feasibility of using Necrosensor in the fat body (Fig. 3C).

We also examined whether Necrosensor can function in the adult midgut, where detection of cell death is empirically challenging. Because of the difficulty to detect cell death in the adult gut, sytox, which is not compatible with fixation, has been used in the gut field (Kolahgar et al., 2015; Liang et al., 2017). We induced cell death by feeding flies with bleomycin, which causes DNA damage in the gut cells (Obata et al., 2018). Necrosensor could detect bleomycin-induced necrotic cells in both the tissue layer and the gut lumen (Fig. 3D–F), demonstrating that it can be a useful tool to detect cell death in the adult midgut.

**Fig. 4.**
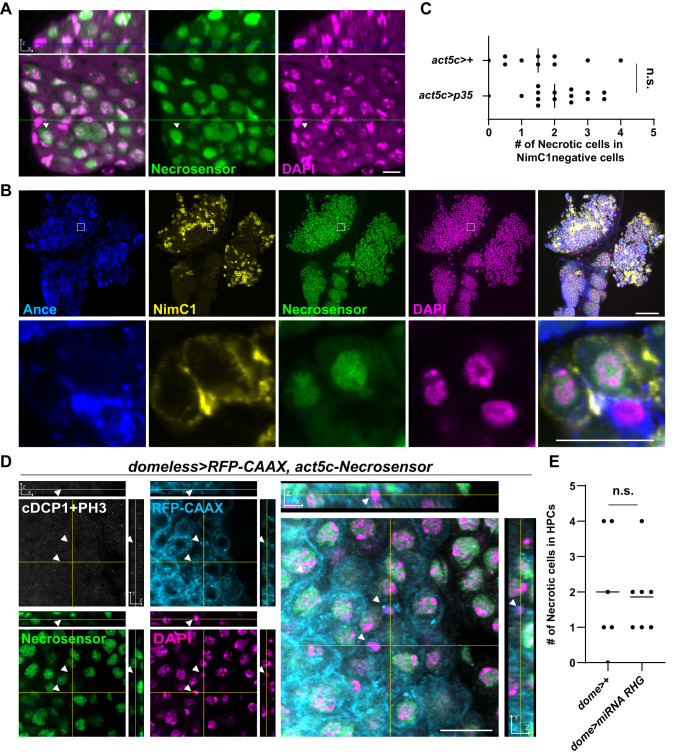
**Necrosensor detects physiological necrosis that occurs during development.** (A) Necrosensor detects previously identified necrosis of germ cells in the male testis. (B) Necrosensor reveals previously unidentified necrosis of hemocyte progenitor cells. (Ance positive, NimC1 negative, indicated by arrowheads) in the lymph gland of developing larvae. The areas in the white rectangles are shown magnified below. (C) p35 expression fails to inhibit necrosis of hemocyte progenitors in the lymph gland (n.s., *P*>0.05, two-tailed unpaired *t*-test). NimC1 (a plasmatocyte marker) is used to distinguish progenitor cells and differentiated cells. (D) The plasma membrane of hematopoietic progenitor cells is marked by RFP-CAAX. cDCP1-/PH3- necrotic cells are observed within the progenitor population (arrowheads). (E) miRNA for *rpr/hid/grim* was expressed in hemocytes of the lymph gland using domeless-Gal4. Apoptosis inhibition by microRNA for *rpr/hid/grim* does not suppress necrosis in hematopoietic progenitor cells. Two-tailed unpaired *t*-test. Scale bars: 10 μm (A); 100 μm (B: top); 100 μm (B: bottom); 10 μm (D).

We also investigated whether Necrosensor can detect physiological necrosis, which occurs during normal development, besides in larval imaginal discs. One example of physiological necrosis is the one that occurs during spermatogenesis in the male testis (Napoletano et al., 2017; Yacobi-Sharon et al., 2013). Previously, PI was used to detect necrotic cells during spermatogenesis. The PI staining required overnight incubation of the live testis (Napoletano et al., 2017), which could potentially confound interpretation of the results. We found germline cells that had lost Necrosensor (Fig. 4A). These data imply that Necrosensor can potentially detect necrosis in a physiological context, although there is a possibility that these cells may have lost Necrosensor due to other mechanisms rather than necrosis. Thus far, we have shown the usefulness of Necrosensor using conditions in which necrosis is already known to occur.

In order to discover previously unidentified cellular necrosis, we interrogated whether a new cell death event can be detected by Necrosensor in tissues and organs where physiological necrosis has not been described. We decided to focus on hematopoietic progenitor cells in the lymph gland, which functions as a hematopoietic organ during larval development. Proliferation of hematopoietic progenitor cells and their differentiation to mature hemocytes, including plasmatocytes and crystal cells, have been intensely studied (Banerjee et al., 2019; Fuwa et al., 2015). But, whether physiological death of the progenitor cells occurs in the lymph gland during development has not been studied to the best of our knowledge. We reasoned that since massive proliferation and differentiation occur, cell death that can be detected by Necrosensor may occur among the hematopoietic progenitors in the lymph gland.

Examining the lymph gland using the Necrosensor revealed that several cells in the medullary zone of the primary lobe do not have the signal of Necrosensor (Fig. S4A). We found that several hemocyte progenitors, which are labeled by a stem cell marker Ance (Benmimoun et al., 2012) but not by the plasmatocyte marker NimrodC1 (Honti et al., 2013), lose signals of Necrosensor (Fig. 4B) in the primary lobe, indicating that physiological necrosis occurs in the premature hematopoietic progenitors. This necrosis of hemocyte progenitors in the lymph gland could not be prevented by *p35* expression (Fig. 4C; Fig. S4B), which inhibits executioner caspases (Hay et al., 1994; Yoo et al., 2002). Importantly, *p35* expression induced the phenotypes that are known to occur by inhibition of caspases: wider width between the midline bristles on the thorax (Levayer et al., 2016) (Fig. S4C), an increase in the number of the progenitor cells in the midgut (Reiff et al., 2019) (Fig. S4D) and prevention of rpr-induced apoptosis in the wing disc (Fig. S4E). This indicates that *p35* that we expressed is functional. Taken together, necrosis of hemocyte progenitors detected by Necrosensor in the lymph gland is not secondary necrosis following traditional apoptosis. Rather, this is a physiological necrosis, which cannot be suppressed by caspase inhibition through p35.

Since this finding of new death in the lymph gland is novel, we paid a special attention on it. We noted that HMGB1 can relocate to the cytoplasm during mitosis (Jia et al., 2020). We also found that relocation of Necrosensor occurred in PH3+ mitotic cells of wing imaginal discs (Fig. S5A). Although Necrosensor stays in the cytoplasm during mitosis and completely disappears from the cytoplasm during necrosis (Fig. S5B) and the phenotypes are readily distinguishable, due to the novelty of discovering progenitor cell necrosis in the lymph gland, we decided to take extra care to prove that this is truly necrosis. For this purpose, we performed triple staining of PH3/cDcp1/Necrosensor. We expressed RFP-CAAX by *domeless-gal4*, hematopoietic progenitor specific Gal4 driver (Banerjee et al., 2019; Fuwa et al., 2015; Honti et al., 2013), to visualize the hematopoietic progenitors. We found that RFP+/PH3-/cDCP1- cells lost Necrosensor (Fig. 4D), indicating that necrosis occurs in hematopoietic progenitors during development. This physiological necrosis occurred even with caspase inhibition by expression of miRNA for *rpr/hid/grim* (Siegrist et al., 2010) (Fig. 4E), indicating that this is not secondary necrosis. We also note that some PH3+ cells demonstrate relocation of Necrosensor to the cytoplasm without its complete disappearance (Fig. S5C), suggesting importance of careful observation of Necrosensor signals.

While we were characterizing Necrosensor, we noticed its potential caveat. When we observed necrotic cells of the hematopoietic organs, we found that cells that formed the dorsal vessel always lose Necrosensor (Fig. 4B). Since all the nuclei of the dorsal vessel cells lost the Necrosensor, it was difficult to consider that all the cells were necrotic. Expression of the *Necrosensor* is driven by the *Act5c* enhancer, which is active in most cell types of *Drosophila*. However, since Necrosensor is based on negative signals, if cells do not express it, it is impossible to tell whether negative signals are due to necrosis or non-expression of the probe. This prompted us to develop an improved reporter, Necrosensor 2.

**Fig. 5.**
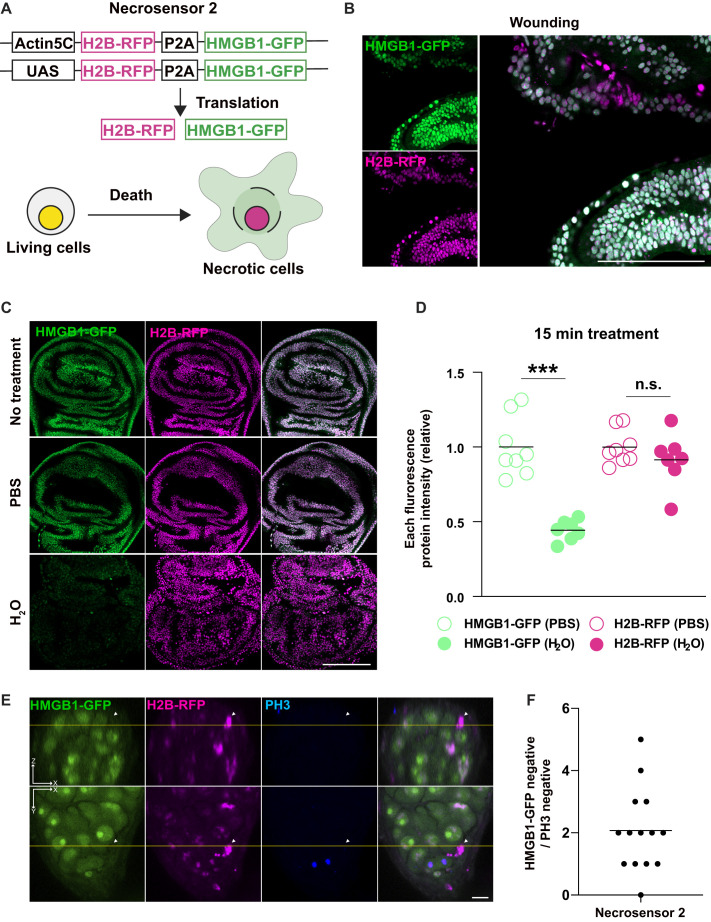
**Development of Necrosensor 2.** (A) A design of Necrosensor 2. Necrosensor (HMGB1-GFP) is connected with H2B-RFP via P2A. HMGB1-GFP is released into the extracellular space upon necrotic stimuli, whereas H2B-RFP remains in the nucleus. (B) Necrosensor 2 detects wound-induced necrosis. Only the HMGB1-GFP signal is lost upon wounding. (C,D) Necrosensor 2 detects osmotic stress-induced necrosis. The fluorescence intensity of GFP and RFP was quantified after taking confocal pictures. The H2B-RFP intensity does not change with the osmotic stress while the HMGB1-GFP intensity is significantly decreased. Two-tailed unpaired *t*-test. (E,F) Necrosensor 2 detects necrosis of germ cells in the male testis. The arrowheads indicate a necrotic cell. Scale bars: 50 μm (B); 100 μm (C); 10 μm (E).

To discriminate necrosis from an absence of *Necrosensor* expression, we connected the original Necrosensor with H2B-RFP via P2A. Thus, the same amount of each fluorescent protein is produced in the reporter-expressing cells. As RFP-tagged histone protein localization is insensitive to cell death-inducible stimuli (Fig. 3A), we expected to be able recognize whether *Necrosensor 2* is expressed in cells (Fig. 5A).

We made two versions of *Necrosensor 2* using the *Act5C* enhancer and UAS (Fig. 5A) and examined whether they behaved as we expected, in the contexts of wounding and osmotic stress. As predicted, GFP signals were lost upon necrotic stimuli, but H2B-RFP was retained at the nucleus (Fig. 5B–D). Overexpression of *Necrosensor 2* with nub-Gal4 did not induce any abnormality in the larval wing disc or in the adult wing morphology (Fig. S6A,B), suggesting its non-invasiveness. Furthermore, physiological necrosis in germ cells during spermatogenesis could also be detected by Necrosensor 2 (Fig. 5E,F).

With Necrosensor 2, we re-examined whether necrosis occurs in the cells that constitute the dorsal vessel or the *Act5C* enhancer activity is absent in those cells. Nuclei of the dorsal vessel cells did not exhibit signals of H2B-RFP, despite the strong F-actin staining with phalloidin (Fig. S6C). This indicates that, in dorsal vessel cells, *Necrosensor* was not expressed, rather than all the cells being necrotic.

## DISCUSSION

Here, we report generation of a new genetically encoded biosensor that detects necrosis *in vivo*. To the best of our knowledge, this is the first development of a genetic system that enables necrosis to be detected in any multicellular organism. Until now, the only method to detect necrosis *in vivo* was with vital dyes. However, the use of vital dyes is accompanied by several complications, such as requiring live staining. With Necrosensor reported in this study, such issues can be overcome. Necrosensor enables imaging of fixed tissues, as well as live analysis of necrosis *in vivo* if the live tissues are visible under the microscope.

One caveat of Necrosensor is that it is based on a negative signal of GFP. To overcome this problem, we generated Necrosensor 2, which drives H2B-RFP and HMGB1-GFP simultaneously. We emphasize that even Necrosensor 2 is not perfect, since intracellular degradation of the probe could affect interpretation. Thus, it is important to confirm necrosis using multiple approaches besides Necrosensor and Necrosensor 2.

Although necrosis has traditionally been considered a response to severe damage and stress, it can also occur as physiological events without any external insult in organisms. In *Drosophila*, necrosis regulates developmental processes in the male germline cells (Napoletano et al., 2017; Yacobi-Sharon et al., 2013) as well as the nurse cell death during oogenesis in the female ovary (Bass et al., 2009). Necrosis also occurs in neuroblasts of the *Cdc20/fizzy* mutant (Kuang et al., 2014). In this report, we demonstrated that Necrosensor can detect necrosis of male germ cells during spermatogenesis as well as necrosis of wing disc cells (Figs 2A,B, 4A; Fig. S2A,B).

With Necrosensor, we discovered previously unidentified, potential necrosis of hemocyte progenitors in the lymph gland, which could not be suppressed by *p35 or miRNA RGH* expression (Fig. 4B,C). Previous single-cell RNA transcriptome data revealed that some of the hematopoietic progenitors and dorsal vessel cells express Eiger, a *Drosophila* TNF ligand (Cho et al., 2020). Although it remains unclear whether *Drosophila* possesses molecularly defined necrosis that is similar to necroptosis in mammals, necroptosis in mammals often involve TNF signaling (Conrad et al., 2016; Li et al., 2019). A recent study showed that Eiger induces necrosis when an executioner caspase is inhibited in *Drosophila* (Li et al., 2019). Furthermore, the *Drosophila* homolog of a tumor suppressor protein, Cylindromatosis, one of the essential regulators of necroptosis in mammals, has been shown to regulate JNK activation in Eiger-induced cell death (Xue et al., 2007). We speculate that Eiger secreted from either hematopoietic progenitors or dorsal vessels might be involved in necrosis of the progenitor cells.

Our genetic necrosis biosensor could open up a new avenue of research, such as identification of as yet undiscovered physiological necrosis in diverse tissues in *Drosophila*, and help push forward the field of cell death within multicellular organisms *in vivo*.

## MATERIALS AND METHODS

### Drosophila husbandry

Flies were maintained as previously described (Yoo et al., 2016). The fly food is composed of the following ingredients: 0.8% agar, 10% glucose, 4.5% corn flour, 3.72% dry yeast, 0.4% propionic acid, 0.3% butyl p-hydroxybenzoate.

### Plasmid construction and transgenic generation

The plasmid for UAS-ASIC2ACA was constructed by subcloning human ASIC2A (BNC1) G430C into the pUASTattB plasmid (Bischof et al., 2007). A human ASIC2A cDNA clone (30915358, ThermoFisher Scientific) was used as a template to make the mutant G430C by overlap PCR. Rat HMGB1-GFP (Hoppe et al., 2006; Scaffidi et al., 2002) (a gift from Dr George Hoppe) was subcloned into the pAct-FRT-polyA-FRT-attB plasmid (a gift from Dr Justin Bosch) to construct a plasmid pAct-FRT-polyA-FRT-HMGB1-GFP-attB. Transgenic flies were made using PhiC31 integration (BestGene). In order to remove the FRT cassette from the pAct-FRT-polyA-FRT-HMGB1-GFP in the transgenic flies, they were crossed to hsFLP and L3 larvae were subjected to heatshock (37°C, 30 min). The resulting strain possesses pAct-HMGB1-GFP (Necrosensor).

For Necrosensor 2, humanH2B-RFP-P2A-ratHMGB1-GFP sequence was synthesized by GenScript and was subcloned into the pActattpB plasmid and the pUAS plasmid to construct plasmid pAct-H2B-RFP-HMGB1-GFP and UAS-H2B-RFP-HMGB1-GFP. Transgenic flies were made using PhiC31 integration (BestGene).

### Drosophila stocks

Flies were crossed and raised at 25°C unless otherwise noted. For wild-type controls, Oregon-R (BL4269) was used. The following fly stocks were used in this study:

*UAS-Src42 CA* (a gift from Dr Tian Xu)

*UAS-rpr* (BL5824)

*UAS-miRNA rpr grim and hid*

*UAS-ASIC2ACA* (generated in this report)

*UAS-RFP-CAAX* (a gift from Dr Shigeo Hayashi)

*pAct5c-HMGB1-GFP* (Necrosensor)

*pAct5c-H2B-RFP-P2A-HMGB1-GFP* (Necrosensor 2)

*UAS-H2B-RFP-P2A-HMGB1-GFP* (Necrosensor 2)

*UAS-dcr2; nub-gal4* (BL25754)

*vg-Gal4* (BL6819)

*domeless-gal4* (a gift from Shoko Nishihara)

*cg-Gal4* (a gift from Dr Iswar Hariharan)

*pnr-Gal4* (BL25758)

*esg-Gal4, UAS-GFP, tub-Gal80ts* (a gift from Dr Norbert Perrimon)

*e22c-Gal4, UAS-dsred2-nuc/cyo hs-hid* (a gift from Dr Michael Galko)

*rotund-Gal4, UAS-rpr, tub-Gal80ts* (Smith-Bolton et al., 2009)

*nub-gal4, tub-gal80ts*

*ubi-HisAV-RFP* (Pandey et al., 2005)

*UAS-p35* (Yoo et al., 2016)

*UAS-miRNA rpr grim and hid* (Siegrist et al., 2010)

### Immunofluorescence and confocal imaging

Larval and adult tissues were dissected in PBS, fixed with 4% paraformaldehyde in PBS and washed in 0.1% Triton X-100 in PBS as previously described (Ikegawa et al., 2023). We used the following antibodies and fluorescent dyes: rabbit cleaved *Drosophila* Dcp-1 antibody (1:200-500, #9578, Cell Signaling Technologies), rabbit ANCE antibody (1:1000, a gift from Dr Elwyn Isaac), mouse NimrodC1 antibody (1:30, a gift from Dr Istvan Ando), mouse Hindsight antibody (1:20, DSHB 1G9), 4,6-diamidino-2- phenylindole (1:1000, D9542, SIGMA), TUNEL, AnnexinV-647 and Propidium Iodide (100 nM, 341-07881,Fujifilm), Alexa Fluor™ 647 Phalloidin (1:500, ThermoFisher Scientific, A22287). Fluorescence images were acquired with confocal microscopes (ZeissLSM780, 880). For PI and AnnexinV-647 (ab219919) staining, wing discs in L3 larvae were dissected in PBS, incubated with PI and AnnexinV-647 (1:100) in PBS immediately after dissection, fixed with 4% paraformaldehyde in PBS and washed in 0.1% Triton X-100 in PBS. To remove the mRNA derived PI signal, discs were treated with RNase for 10 min at room temperature.

### Electron Microscopy

To discriminate necrosis, secondary necrosis and apoptosis, overexpression of ASIC2A or rpr was conducted with Gal80ts system. Larvae carrying *nub-gal4, tub-gal80ts* with either *UAS-ASIC2A CA* or *UAS-rpr* were kept at 18°C until L3, when larvae were incubated at 30°C for 8 h. Larvae were inverted in PBS, followed by immediate fixation with 2.5% glutaraldehyde and 2% paraformaldehyde in 0.1 M cacodylate buffer (pH 7.4) at 4°C for overnight. After washing five times with 0.1 M cacodylate buffer, samples were further fixed with 1% osmium tetroxide in 0.1 M cacodylate buffer at 4°C for 1 h and then washed five times with Milli-Q water. Samples were incubated in 1% uranyl acetate at 4°C overnight and washed five times with Milli-Q water. Samples were dehydrated with ethanol (20, 50, 70, 90 and 99.5% for 5 min each and twice in 100% for 10 min) and followed by infiltration with Epon812 resin (TAAB). Polymerization was performed at 60°C for 72 h. Ultrathin sections (60–80 nm) were cut with a diamond knife on an ultramicrotome (Leica EM UC7, Leica Microsystems, Wetzlar, Germany) and placed on a piece of silicon wafer. The sections were stained with uranyl acetate followed by lead nitrate and observed with a field emission scanning electron microscope JSM-7900F (JEOL, Tokyo, Japan) at 7 kV using back scattered electron detector.

#### Detection of physiological cell death in the wing disc with PI and AnnexinV

For PI and AnnexinV-647 (ab29919, abcam) staining, wing discs in L3 larvae were dissected in PBS, incubated with PI and AnnexinV-647 (1:100) in PBS for 10 min, fixed with 4% paraformaldehyde in PBS and washed in 0.1% Triton X-100 in PBS. To remove the mRNA-derived PI signal, discs were treated with RNase (EN0531, ThermoFisher Scientific) for 10 min at a room temperature, followed by washing in 0.1% Triton X-100 in PBS and mounted on a slide glass. The number of cells in each population was counted manually.

#### Hid-induced cell death analysis with TUNEL and cDCP1 antibody

The TUNEL assay was performed using the ApopTag Red *in situ* apoptosis detection kit (Millipore) according to the manufacturer's instructions. Fifteen min after heat shock induction, wing discs were dissected in 1xPBS and fixed in 1xPBS with 4% paraformaldehyde at the room temperature. After fixation, samples were washed with PBS 0.1% Triton-X100 and incubated in equilibration buffer (Apop Tag kit; Millipore) for 10 s. Then, samples were incubated in reaction buffer (TdT enzyme; ratio 7:3; Apop Tag kit) at 37°C for 1 h. The TdT reaction mix was replaced with stop buffer (diluted 1:34 in dH_2_O; Apop Tag kit) and incubated for 10 min at RT. Samples were then washed with PBS 0.1% Triton-X100 three times and incubated with anti-digoxigenin antibody solution (diluted 31:34 in blocking solution; ApopTag kit) and anti-cDCP1 antibody overnight at 4°C. Samples were then washed with PBS 0.1% Triton-X100 three times again and incubated with the secondary antibody for 3 h at room temperature, followed by washing with PBS 0.1% Triton-X100 thee times and mounted on a glass slide.

#### Wounding of wing discs

Wing discs in L3 larvae were dissected out and wounded by tweezers in PBS. Pictures were taken immediately after wounding.

#### Osmotic stress induction

For osmotic stress induction, wandering L3 larvae were inverted and cultured with either PBS or H_2_O for 1, 10, 15, 30 or 60 min.

#### Measurement of secreted HMGB1-GFP

Fat bodies from five larvae were dissected out in PBS and incubated in either 50 μl PBS or H_2_O. To measure the HMGB1-GFP intensity secreted from fat bodies, we collected 1 μl supernatant and used the nanodrop 3000 to measure the GFP intensity in the supernatant. The fluorescence intensity at the emission wavelength of 519 nm was measured.

#### Validation of Necrosensor 2

To observe the Necrosensor 2 behavior upon wounding, wing discs were dissected in PBS and wounded by forceps. Immediately after wounding, discs were fixed and observed by confocal microscopy. To observe the Necrosensor 2 behavior upon H_2_O treatment, discs were dissected in PBS and incubated in either PBS or H_2_O for 15 min, followed by fixation. HMGB1-GFP and H2B-RFP were detected by confocal microscopy and intensities of each fluorescence were quantified.

#### X-ray irradiation

For irradiation experiments, wandering L3 larvae were irradiated by 60 Gy X-ray with the MX-160 Labo (mediXtec). Three hours after irradiation, wing discs were dissected and fixed.

#### Hid expression with heat shock

Ectopic expression of *hid* was induced by heat shock using hs-hid. Wandering larvae (*CyO, hs-hid/+; act5c-Necrosensor/+*) were incubated in a 37°C water bath for 5 min and wing discs were dissected in PBS at 3 h after the heat shock.

#### Laser-inflicted damage and live imaging of embryonic cells

Embryos laid by Necrosensor/His2Av-RFP female flies were incubated at 25°C, devitellinized and mounted on the ventral side to image on the dorsal side for IR laser inflicted damage and two-photon time-lapse imaging using an Olympus Fluoview FVMPE-RS system equipped with an InSight DeepSee pulsed IR Dual-Line laser system (Spectra Physics) using a 25× water immersion objective (N.A.=1.05). For laser-inflicted damage, the tunable laser line was tuned at 930 nm. With a power set to 15% (corresponding to 72 mW measured approximately 0.2 cm away from the objective; Thorlabs, PM16-121), the beam was scanned at a speed of 7.111 μs/μm with repetitions for a duration of 1 s along a line of 28.125 μm in length. The line was chosen to be at the center of the dorsal embryonic surface, parallel to the anterior-posterior axis and at about 15 μm beneath the vitelline membrane. Live imaging was performed with 930 and 1040 nm wavelength light for HMGB1-GFP and His2Av-RFP, respectively. This procedure was repeated in five embryos during the stage of cellularization.

#### Bleomycin treatment of adult flies

For bleomycin treatment, newly eclosed male flies (*Act5c-Necrosensor/+*) were collected for 2 days and transferred to the food containing 200 μM bleomycin (B3972, Tokyo Chemical Industry). Flies were fed with bleomycin for 24 h, then dissected in PBS, fixed with 4% paraformaldehyde in PBS and washed in 0.1% Triton X-100 in PBS as previously described (Sasaki et al., 2021).

#### p35 expression

p35 was expressed in the whole body (*act-Gal4*), in the thorax (*pnr-Gal4*), in the gut progenitors (*esg-Gal4, tub-Gal80ts*) and in the wing pouch (*rotun-Gal4, UAS-rpr, tub- Gal80ts*) by crossing UAS-p35 to respective Gal4 lines. Measurement of the width between central microchaetae was performed in 1–2-day-old female flies using SMZ18 stereomicroscope (Nikon). Expression of p35 in the gut progenitors was induced by incubating flies at 30°C for 7 days (before induction, flies were kept at 18°C) as performed in (Sasaki et al., 2021). Expression of *reaper* and *p35* in the wing pouch was initiated by shifting the temperature from 18°C to 30°C on day 4 after egg laying and was kept on for 3 days.

#### Statistical analysis

Statistical tests used were indicated in the figure captions. Sample sizes were determined empirically based on the observed effects. All the statistical analyses were performed using GraphPad Prism 9. Statistical significance is shown by asterisk; *P<0.05, **P≦0.01, ***P≦0.001, ****P≦0.0001.

## Supplementary Material

10.1242/biolopen.060104_sup1 Supplementary information
