# Supplementary material for: Necrosensor: a genetically encoded fluorescent sensor for visualizing necrosis in Drosophila
Source: Biol Open. 2024 Jan 22;13(1):bio060104. doi: 10.1242/bio.060104 (PMC10836653; doi:10.1242/bio.060104)
Supplement: Supplementary information [file biolopen-13-060104-s1.pdf]

Figure S1

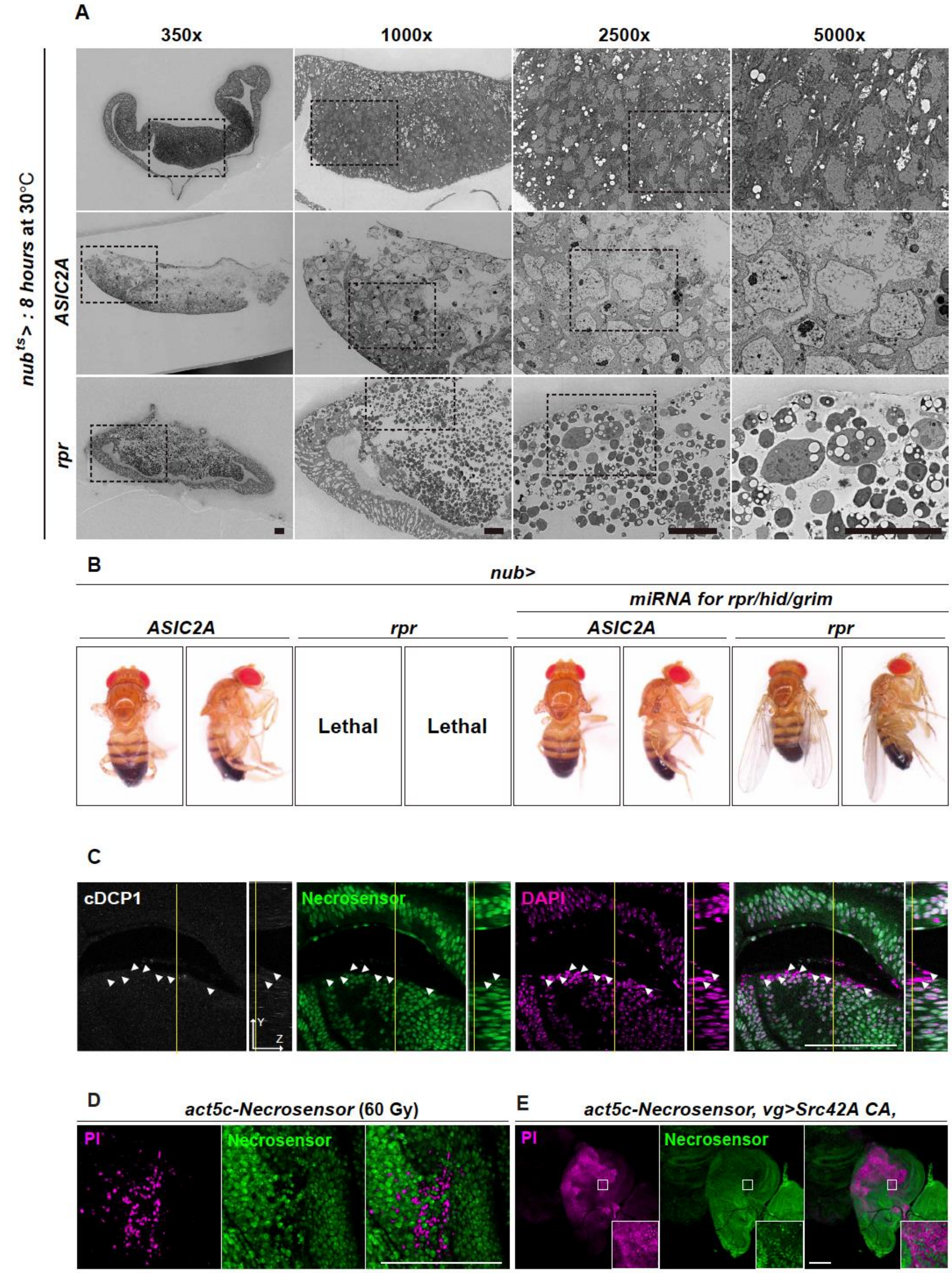

Biology Open • Supplementary information

Fig. S1.

A. EM images show that ASIC2A-induced cell death does not have apoptotic features such as chromatin condensation or apoptotic bodies.

B. *microRNA for rpr/hid/grim* does not suppress ASIC2A-induced wing loss.

C. Wounding induces necrosis without activation of caspase (arrowheads).

D. Necrosensor detects X-ray-induced necrosis.

E. Necrosensor is lost upon Src42A CA overexpression

Scale bars, 10 μm (A), 100 μm (C-E)

Figure S2

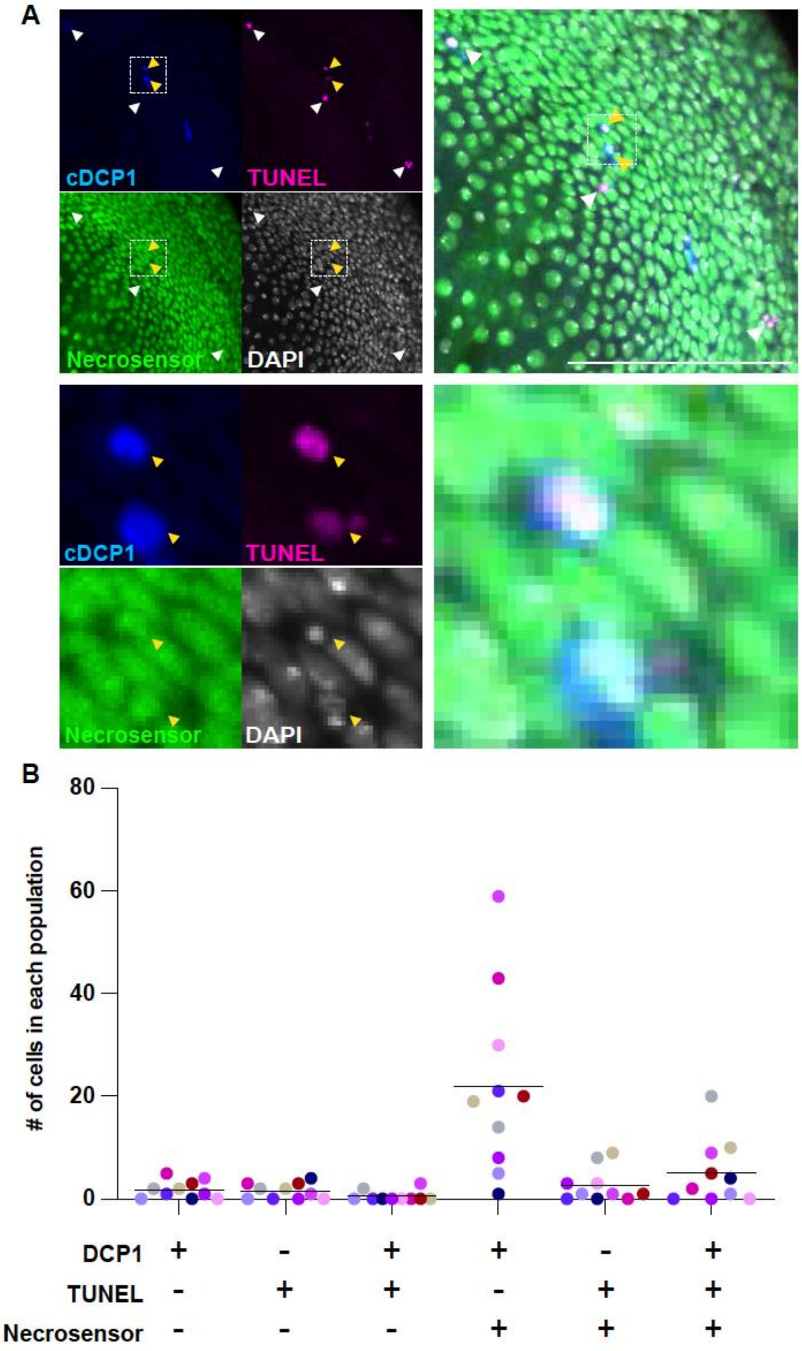

Fig. S2.

A. anti-cDCP1 and TUNEL staining detect physiological cell death in the wing disc. A majority of apoptotic marker positive cells do not lose Necrosensor (yellow and white arrowheads indicate cDCP1+, TUNEL+ cells and cDCP1-, TUNEL+ cells respectively).

B. Physiologically dying cells are categorized into six groups. Each dot represents the number of categorized cells. Ten independent wing discs were used for the analysis and the same color indicates the same wing disc.

Scale bars, 50 μm (A)

Figure S3

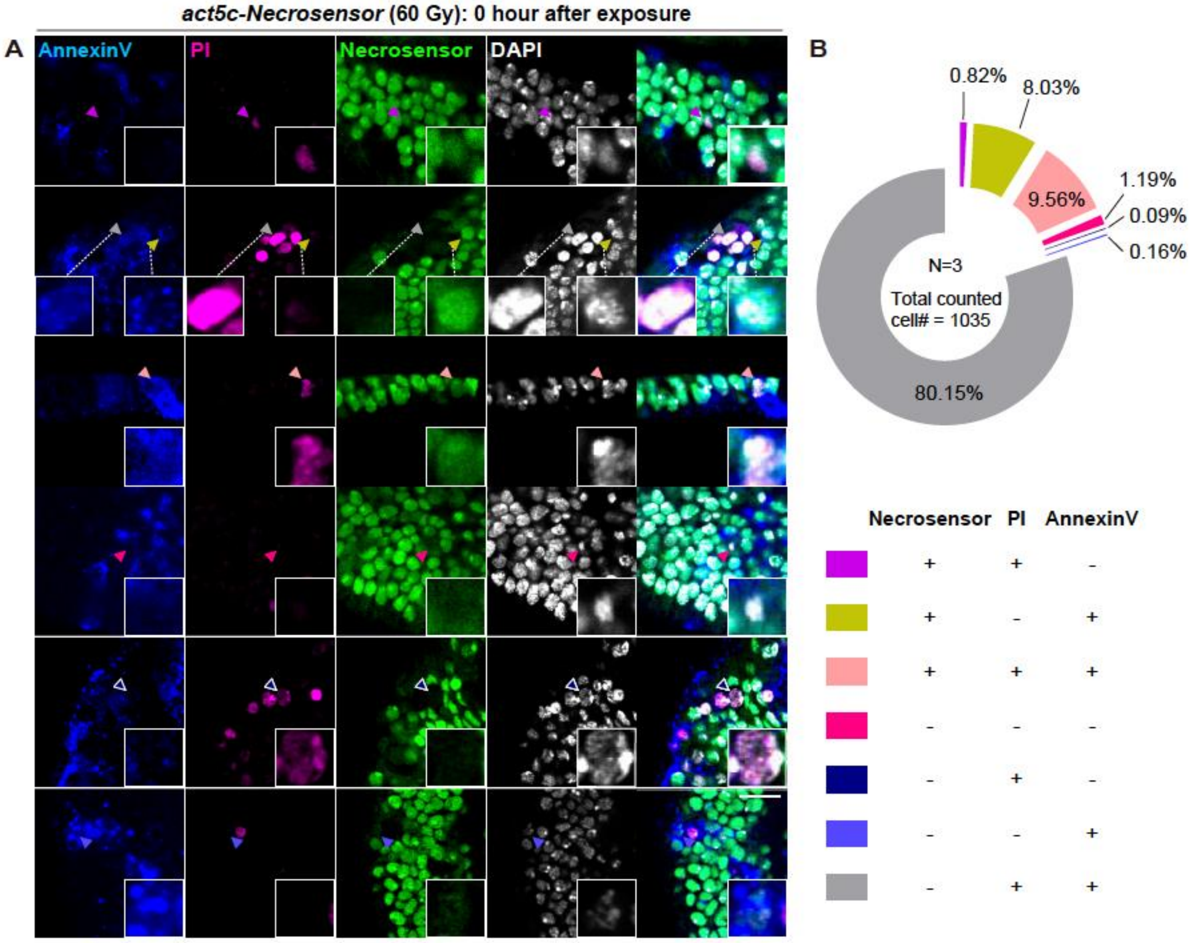

Fig. S3.

A-B. Larvae expressing Necrosensor were exposed to X-ray. Dissected wing discs were stained with Annexin V and PI to detect X-ray-induced cell death. A majority of PI+, Annexin V+ necrotic cells lose Necrosensor.

Scale bars, 10 μm (A)

Biology Open • Supplementary information

Figure S4

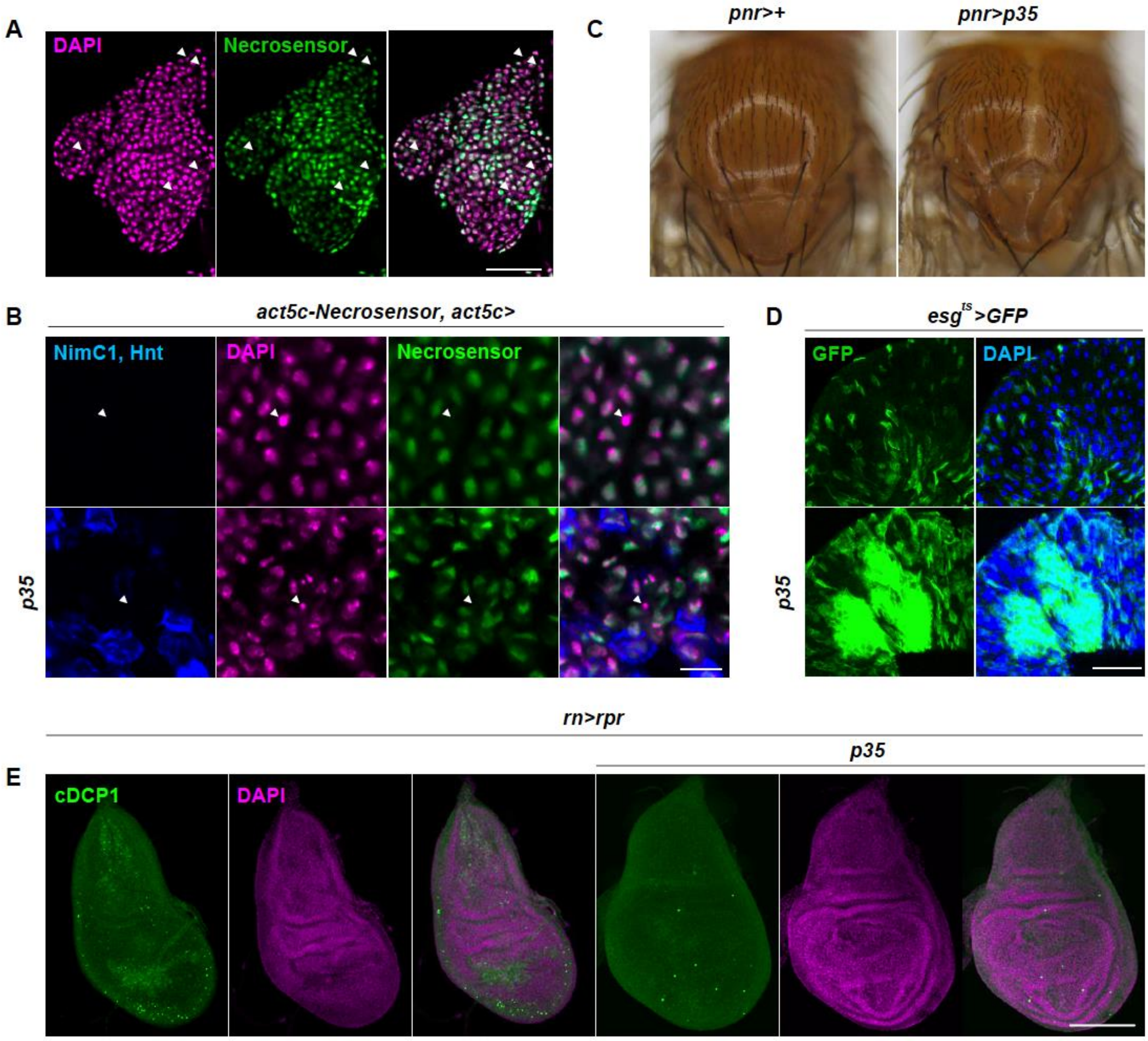

Fig. S4.

A. There are several cells without Necrosenosr signals in the primary lobes of the lymph gland. Arrowheads indicate cells without Necrosensor signals.

B. Representative pictures of hemocyte progenitors (NimC1 negative, Hindsight negative, indicated by arrowheads) with/without p35 expression. NimC1 (a plasmatocyte marker) and Hindsight (a crystal cell marker) were used to label differentiated cells.

C. p35 expression in the midline of the notum makes the width between central microchaetae wider.

D. p35 expression in the gut progenitors induces an increase of their number.

E. p35 expression in the wing pouch suppresses rpr-mediated caspase activation.

Scale bars, 100 μm (A, E), 10 μm (B), 50 μm (D)

Biology Open • Supplementary information

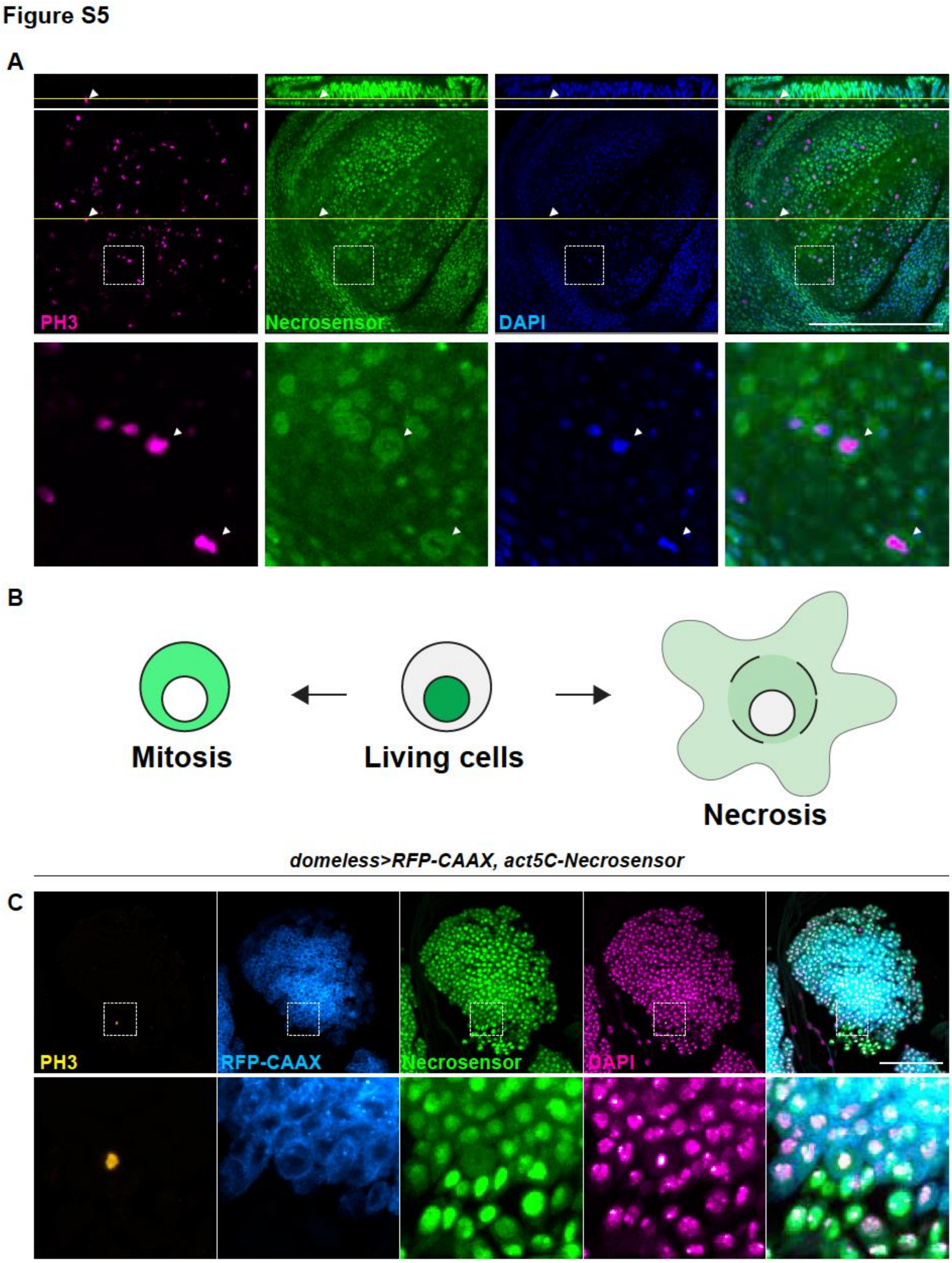

Fig. S5.

A-B. Necrosensor relocates to the cytoplasm in mitotic cells since the nuclear membrane is reorganized during mitosis.

C. Necrosensor relocates to the cytoplasm in mitotic hematopoietic progenitors.

Scale bars, 100 μm (A, C)

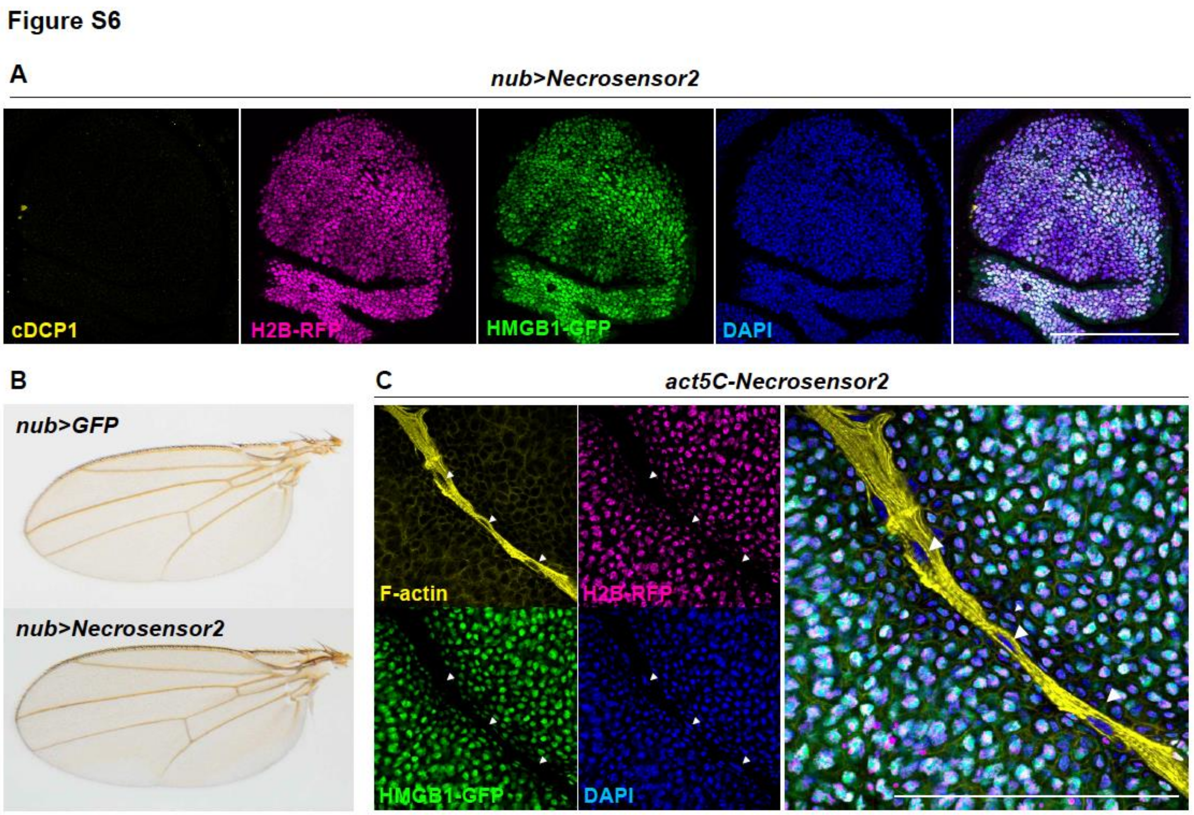

Fig. S6.

A-B. Overexpression of Necrosensor 2 in the wing pouch neither activates caspase nor impairs the wing development.

C. The dorsal aorta (arrow heads) does not express Necrosensor, indicating that the Actin5c enhancer is inactive in the dorsal aorta.

Scale bars, 100 μm (A, C)
